# Flickering nanometre-scale disorder in a crystal lattice tracked by plasmonic flare light emission

**DOI:** 10.1038/s41467-019-14150-w

**Published:** 2020-02-03

**Authors:** Cloudy Carnegie, Mattin Urbieta, Rohit Chikkaraddy, Bart de Nijs, Jack Griffiths, William M. Deacon, Marlous Kamp, Nerea Zabala, Javier Aizpurua, Jeremy J. Baumberg

**Affiliations:** 1 Department of Physics, NanoPhotonics Centre, Cavendish Laboratory, University of Cambridge, JJ Thompson Avenue, Cambridge, CB3 0HE UK; 2 Department of Electricity and Electronics, FCT/ZTF, University of the Basque Country UPV/EHU, 48080 Bilbao, Spain; 3 Materials Physics Center CSIC-UPV/EHU and Donostia International Physics Center DIPC, Paseo Manuel de Lardizabal, 20018 Donostia-San Sebastián, Spain

**Keywords:** Electronic devices, Nanoparticles, Nanophotonics and plasmonics, Nanocavities

## Abstract

The dynamic restructuring of metal nanoparticle surfaces is known to greatly influence their catalytic, electronic transport, and chemical binding functionalities. Here we show for the first time that non-equilibrium atomic-scale lattice defects can be detected in nanoparticles by purely optical means. These fluctuating states determine interface electronic transport for molecular electronics but because such rearrangements are low energy, measuring their rapid dynamics on single nanostructures by X-rays, electron beams, or tunnelling microscopies, is invasive and damaging. We utilise nano-optics at the sub-5nm scale to reveal rapid (on the millisecond timescale) evolution of defect morphologies on facets of gold nanoparticles on a mirror. Besides dynamic structural information, this highlights fundamental questions about defining bulk plasma frequencies for metals probed at the nanoscale.

## Abstract (web summary)

Dynamic restructuring of metal nanoparticle surfaces greatly influences their catalytic, electronic transport, and chemical binding functionalities. Here, the authors show that non-equilibrium atomic-scale lattice defects can be detected in nanoparticles by using nano-optics at the sub-5nm scale.

## Introduction

The ability to observe atomic restructuring processes at metallic surfaces has been an intense focus for understanding catalysis^1–3^, material hardness and fracture^4,5^, surface wetting^6^, as well as interface phenomena^7,8^ within molecular electronics^9^, spintronics and magnetic storage devices^6^. Nanomaterials and nanoparticles offer emergent tunable properties^10,11^ as they are composed of large fractions of surface atoms, while understanding molecular binding onto different crystalline facets is at the root of catalytic activity, electrochemical processes and surface restructurings. Well-established techniques to study such effects have increasingly shifted from X-ray^12^, electron-scattering^2,13^ and neutron-scattering on large areas^14^, to tracking of individual atomic facets with transmission electron- and scanning tunnelling-microscopies^15,16^. However these are problematic to use at higher resolution at room temperature and ambient conditions, with strong molecular interactions causing fast dynamics and restructuring^13^. It has long been thought impossible to study such systems optically and non-invasively, due to the Rayleigh wavelength resolution and sensitivity issues.

Using the recent discovery that plasmonics allows light to be trapped in ‘hot-spots’ on the atomic scale^17–19^, we show here that optics is capable of studying the millisecond dynamics of crystal defects with a resolution of a few nm. We reveal a new phenomenon in metallic gold nanoparticles, which suggests restructuring at transient defects such as twin planes which lead to localised changes in plasma frequency, and that likely accounts for much of the longstanding inconsistencies around the complex optical permittivity of noble metal surfaces at room temperature^20–23^. Strong coloured flares of light are briefly emitted, with their spectra independent of surface molecule, instead arising from light penetrating into the metal. We show that gold atom dynamics driven by localised optically-induced forces depends on molecular surface binding, and show that light injected into the metal from such tightly-confined modes induces nonlinear optical effects even for incoherent illumination. Resolving such flickering disorder has implications for many surface phenomena which average over this behaviour such as photocatalysis at nanoparticles, tip-enhanced scanning spectroscopies, hot electron emission, and others.

## Results

### Plasmonic nanocavity assembly

**Fig. 1:**
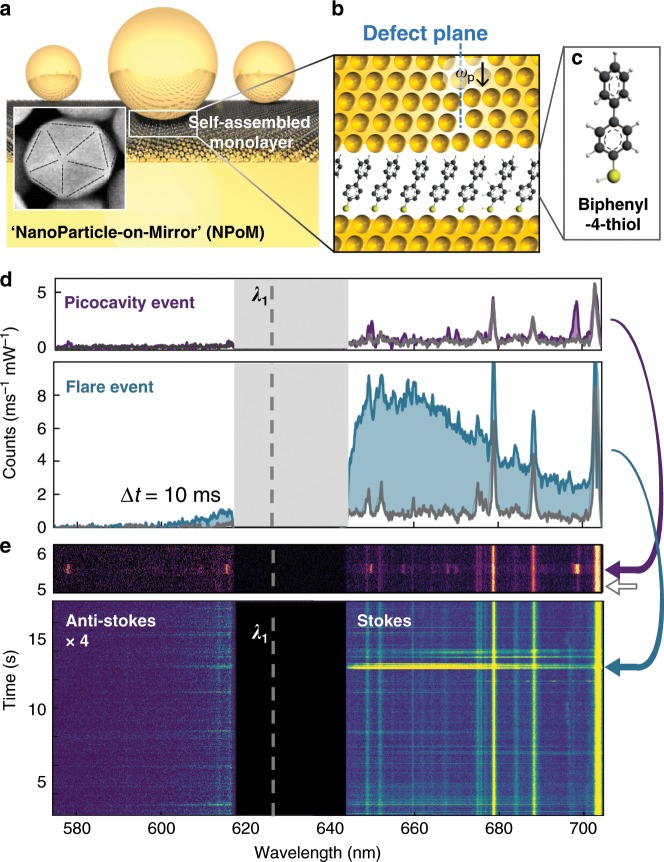
Flares and picocavity transient events in plasmonic nanocavities. **a** Nanoparticle-on-Mirror construct, sandwiching a molecular monolayer. Inset shows typical icosahedral nanoparticle, with twin planes dashed. **b** Schematic transient defect boundary (dashed) at surface facet of nanoparticle. **c** Biphenyl-4-thiol (BPT) molecule making up monolayer (for others see below). **d** Inelastic scattering spectra; top panel shows picocavity event (purple), bottom panel shows broad flare spectrum (blue), each compared to stable BPT spectra from same nanoparticle (grey). **e** Kinetic SERS spectra showing time dynamics of picocavity spectra (top panel, purple arrow) and multiple flare spectra (bottom panel, blue arrow identifies flare spectrum in **d**).

We study thousands of individual nanoparticles on each nano-fabricated sample, using automated nanoparticle location to collect statistics on millions of events^24^. Individual gold nanoparticles are spaced above an ultra-flat gold film by a self-assembled molecular monolayer (SAM) (initially biphenyl-4-thiol, BPT) forming a plasmonic hotspot in the resulting 1.4 nm gap between mirror and nanoparticle (Fig. 1a–c, see Methods section). These nanoparticle-on-mirror (NPoM) constructs of near-spherical 80 nm diameter colloidal noble-metal particles formed of cubic single-crystals are however not quite uniform, most commonly appearing as icosahedrons and cuboctahedrons (as well as less spherical pentagonal bipyramids and flat prisms which have different scattering spectral signatures and are avoided here)^25^. The lowest coupled-plasmon mode is red-shifted into the near-infrared depending on the lower facet area and nanoparticle diameter, as well as the gap size and contents. However dark-field scattering on each NPoM shows these coupled plasmon modes lie within a narrow distribution, *λ*_*C*_ = 800 ± 20 nm (Supplementary Figs. 1 and 2) as predicted from the average gap size and composition^26,27^, showing the robust reliable nano-construct formation.

All such nanoparticles contain defects and twin boundaries which have previously been considered as scattering centres for electrons^14^, but which we show here also affect the local metal permittivity and therefore the plasma frequency in their immediate vicinity. Because such plasmonic nanocavities confine light so tightly (to <10 nm lengthscales^17,27^), they become directly sensitive to such defects. While the effects of Au adatoms are seen in UHV at low temperatures in scanning tunnelling microscopy (STM)^28,29^, as well as recently in the optics^18^ of plasmonic gaps, defect planes (Fig. 1b) are also prevalent in these nanoparticles^30^. Even at temperatures well below their melting point, nanoparticles can fluctuate between different microstructures^31^, while detailed quasi-molten models indicate near-surface atoms are much more mobile than bulk atoms. Locally depositing additional energy (by laser irradiation) into these plasmonic nanocavities can create or move defects across the nanoparticle surface, leading to fleeting changes in local environment around the hotspot.

### Appearance of flare emission

Here, we show a new class of optical transients seen in both Rayleigh-scattering and Raman-scattering which directly capture nm-scale properties from local defects in the crystal structure of the metal. Incident laser light of 100–700 µW at 633 nm is filtered out from the scattered light to reveal inelastic scattering on 10 ms timescales, which exceeds 10^3^ integrated counts.mW^−1^.ms^−1^ because of the intense nanocavity field confinement^27^ (Fig. 1d, e). Sharp lines from surface-enhanced Raman scattering (SERS) of the BPT vibrational modes can be clearly seen on the Stokes side of the spectra, with the large SERS enhancements enabling short integration times, thus accessing short-lived spectral features. For the few hundred BPT molecules inside the coupled-plasmon gap mode volume^32^, their close-packing in the SAM ensures fixed-energy stable ‘persistent’ SERS lines are seen. Using the anti-Stokes-to-Stokes ratio, the molecular temperature is estimated as 341 ± 17 K, although the electronic Raman scattering (ERS) background contribution suggests the electrons in the metal might be up to 300 K warmer in these pumped conditions (Supplementary Fig. 3).

Aside from these persistent lines, two other classes of spectral feature are of interest. The first (top panels Fig. 1d, e) are new vibrational lines appearing and disappearing, which evidence ‘picocavity’ formation. Previously discussed in detail at cryogenic and room temperatures^18,24^, these arise from gold adatoms pulled out of the gap surface facets by trapped light, leading to extra optical confinement and the breaking of Raman selection rules^18,33^, that gives single molecule SERS. Instead here we concentrate on rarer but more intense spectrally-broad features (bottom panels Fig. 1d, e, Supplementary Fig. 4) termed ‘flares’. We emphasise that such features can be seen in published data^34–36^ over the last decades, but have not been investigated. In order to methodically analyse these spectral events, our automated experiments capture over a million time-dependent scattering spectra for >3000 NPoMs.

**Fig. 2:**
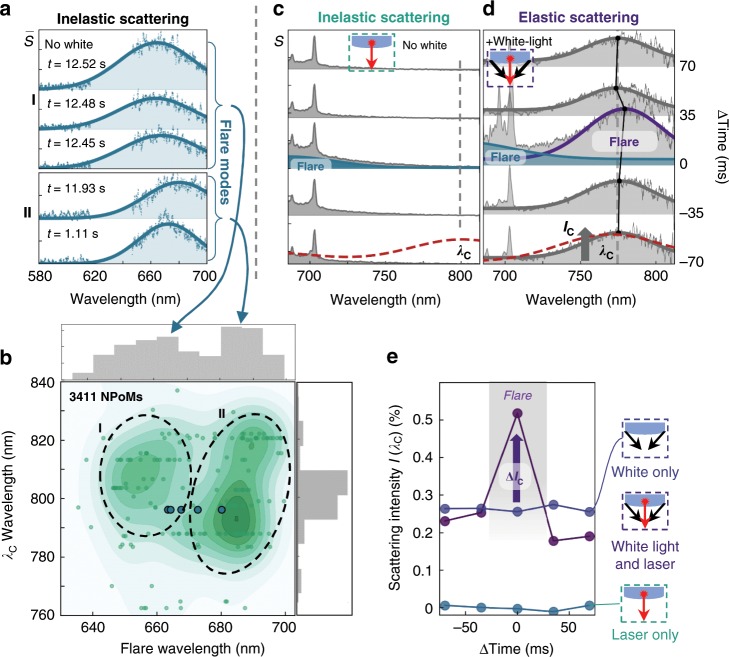
Spectral characteristics of flare events. **a** Different flare inelastic scattering spectra (with persistent SERS lines subtracted, $\bar S$) from a single NPoM, collected at different times *t*. Spectra sorted into groups I,II by emission wavelength. **b** Mapping plasmon coupled-mode wavelength *λ*_*C*_ against flare mode centre wavelength *λ*_*f*_ for flares from 3411 NPoMs. Two distinct groups are identified, grey histograms show distributions. Points corresponding to spectra in **a** are circled. **c, d** Time-dependent raw scattering spectra for longer-wavelength spectral window, (**c**) taken with laser only, or (**d**) with white-light and 633 nm laser irradiation simultaneously. Initial *λ*_*C*_ mode position (black line) transiently redshifts during flare (purple curve) at Δ*t* = 0, blue curves show fit to flare beneath SERS lines. For laser alone, no coupled mode is visible (dashed line shows *λ*_*C*_ mode taken with white light prior to kinetic series). **e** Detected intensity *I*(*λ*_*C*_) from (**c, d**) normalised to Lambertian scatter, extracted around flare event. Additional flare emission (Δ*I*_*C*_) only seen with both laser and white-light illumination (purple).

To better identify the features of these events, the initial spectrum in each time series is subtracted from all subsequent spectra, giving only the intensity increases for brief flare events (picocavity events are filtered out). Examples from a kinetic series on a single NPoM tracked over 35 s demonstrate variations in width and spectral position of several transient flares occurring briefly at different times (Fig. 2a). The lack of sharp features on these spectra imply that the SERS of the molecular vibrations does not change during flaring, emphasising their different origin to picocavities and their emergence from electronic Raman scattering inside the metal itself. The flare spectra can be well fit to Gaussian lineshapes, yielding emission also at shorter wavelengths than the incident laser (below 620 nm) (Supplementary Fig. 5).

To survey this range of flares, a map of the fitted flare wavelength vs. nanoparticle coupled mode position is plotted for all NPoMs (876 flares, Fig. 2b). Although the coupled mode positions lie at $\lambda _C \simeq 800$ nm, there is a bi-modal distribution in flare wavelengths giving information not unpacked in standard scattering experiments. We label the two cluster groups I and II (Fig. 2a, b) and note that even a single NPoM construct can show flare events of both types within a few seconds. With only laser irradiation, inelastic emission is negligible around *λ*_*C*_ during flares, as well as other times (Fig. 2c, which shows time series of inelastic scattering every 10 ms with a single flare event as indicated shaded).

Besides this inelastic light emission, elastic (Rayleigh) dark-field scattering gives further information about morphological changes to NPoM constructs^27^, particularly at the coupled-mode spectral peak *λ*_*C*_. To investigate what happens during flares, in addition to laser excitation the system is simultaneously irradiated with broadband incoherent white-light and the coupled mode peak observed in real time from the elastic scattering (Fig. 2d, Supplementary Fig. 6). Although this shows minimal long-term shifts after the laser irradiation (Supplementary Fig. 1) confirming there are no long-term changes to the NPoM geometry at these laser powers, it varies during flares (marked with the purple curve in Fig. 2d). In this spectral window, besides higher wavenumber BPT vibrations, the tails of flare events can be seen at the same time as this NPoM coupled mode. During flaring, the coupled mode is seen to give small but clear instantaneous redshifts in the coupled plasmon *λ*_*C*_ (purple curve Fig. 2d) before fully returning to its initial mode position. The scattering intensity *I*(*λ*_*C*_) mode transiently increases through each flare event (Fig. 2e), but only when the NPoM is excited by both laser-light and white-light illumination.

Our data strongly suggest that flares arise from the gold facets cladding the molecules in the gap. Flares do not arise from thermal heating since the emission does not match black-body spectra. Nor are optically-measured temperatures high enough for visible wavelength emission (Supplementary Fig. 3). The lack of changes to the measured molecular SERS strongly constrains changes in geometry, suggesting the nanocavity shape is not significantly perturbed, as also confirmed by the weak spectral shifts in the coupled mode. Molecular fluorescence from these non-resonant molecules is also highly unlikely and as we show below, changing the molecules does not change the flare spectra. Flares are thus ubiquitous in nanocavity plasmonics, seen for a wide range in NP sizes, gaps, and spacers, but only clearly revealed for fast SERS measurements when such transients are resolved.

### Simulations and simple model

**Fig. 3:**
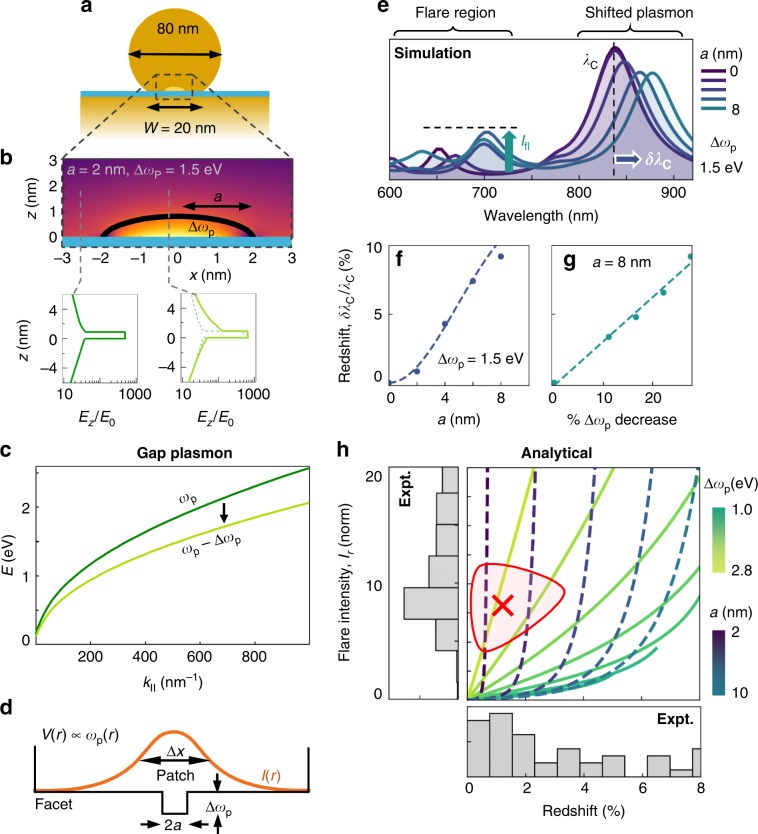
Simulation of flares. **a** BEM simulation geometry (details in SI) with (**b**) region of defect patch of lower *ω*_*p*_ and radius *a* (bounded by black line) giving optical field inside patch at coupled mode resonance *λ*_*C*_ (*E*_*z*_ slices across gap inside & outside the patch shown below). **c** Dispersion relation for gap plasmon with 20% reduction in local *ω*_*p*_. **d** Analytic model for defect, reducing the effective *ω*_*p*_ within a central patch of the nanoparticle facet. I(r) is intensity of the coupled mode in the gap. **e** Simulated scattering cross section for decrease Δ*ω*_*p*_ = 1.5 eV and *a* = 0–8 nm, showing flare intensity $I_{{\mathrm{fl}}}$ and mode redshift $\delta \lambda _C$. **f, g** Simulated (points) and analytical (dashed lines) coupled mode redshifts vs*. a* and Δ*ω*_*p*_, showing their agreement. **h** Analytical model for relative flare intensity vs. mode redshift for (dashed) *a* = 2, 4, 6, 8, 10 nm over Δ*ω*_*p*_ = 0–3.5 eV and (solid) Δ*ω*_*p*_ = 1, 1.3, 1.6, 1.9, 2.2, 2.5, 2.8 eV over *a* = 0–15 nm. Grey histograms show experimental measurements of flare intensity (left) and *λ*_*C*_ mode redshift (bottom). Red marker shows maximum likelihood realisation.

We now present a model which can explain these observations, and is based on local changes in the plasmon frequency of gold near localised transient defects, that enhances light penetrating into the metal and hence ERS emission. Anomalous absorptivity measurements on high reflectivity metals have led to suggestions that grain boundaries and lattice defects might reduce the local electron density *n*_e_, by as much as four-fold^37^ (Fig. 1b), which would reduce the plasma frequency $\omega _{\mathrm{p}} \propto \sqrt {n_e}$ by two-fold. Defects distort the band structure, changing also the effective electron mass m^*^ and thus also changing *ω*_*p*_. To model the influence of such effects, geometries with a surface defect on one facet (Fig. 3a) are considered in boundary element method (BEM) simulations^38,39^ (Supplementary Note 1). We start with small patches of radius *a* (smaller than the facet width *w* = 20 nm) and effective local plasma frequency $\omega _p - {\mathrm{\Delta }}\omega _p$, lower than that of the bulk gold of the nanoparticle (although other geometries explored include cracks, voids, bowing, and rounding, see Supplementary Fig. 7–13). Results are not affected by the patch heights (Supplementary Fig. 8b) due to the short <2 nm penetration depth of light inside the metal (as seen in the near-field optical field distribution at *λ*_*C*_, Fig. 3b). Gap plasmons thus probe the crystal structure in the vicinity of the facet surfaces. Increased penetration of light at these defects is observed due to continuity of $\varepsilon E_z$ normal to the facet which gives relative field strengths in the metal vs. the gap of $e(\omega _p) = |\varepsilon _g/\varepsilon _m| = |\varepsilon _g/\left( {\varepsilon _\infty - \omega _p^2/\omega ^2} \right)|$ that increase at lower *ω*_*p*_ (the background permittivity $\varepsilon _\infty$ remains constant). When the patch size increases (*a* = 0-8 nm) redshifts in *λ*_*C*_ are seen, as well as modes emerging around 600–700 nm (Fig. 3e).

Simple analytic expressions are helpful to allow the experimental data to be used to estimate the effective Δ*ω*_*p*_ and *a*. The redshifts can be intuitively understood from the effect of reducing *ω*_*p*_ on the gap plasmon dispersion, decreasing the energy at each in-plane $k_{||}$ inside the metal-insulator-metal waveguide (Fig. 3c) as $\omega ^2(r) = \omega _p^2\left( r \right)\left[ {\varepsilon _\infty + 2\varepsilon _g/(k_{||}d)} \right]^{ - 1}$ for radial position *r*, gap size *d*, with permittivity $\varepsilon _g$ in the gap^27,40,41^. The waveguide is bounded by the facet of width *w* which sets the allowed in-plane $k_{||}$ for the plasmon cavity modes. The shifted energy for the lowest gap plasmon considered perturbatively (Fig. 3d) thus leads to redshifts (Supplementary Note 2)

1 $$\frac{{\delta \lambda _c}}{{\lambda _c}} = \left( {\frac{{{\mathrm{\Delta }}\omega _p}}{{\omega _p}}} \right)\frac{1}{2}\left\{ {1 - \exp \left( { - \frac{{\varepsilon _ga^2\ln 2}}{{2Rd}}} \right)} \right\}$$

for nanoparticle radius *R*. This dependence agrees very well with the full simulations (Fig. 3f, g).

We now explain the flare emission observed in Figs. 1d, 2a by considering electronic Raman scattering (ERS) inside the metal at these defects. In the ERS process, electrons photo-excited to a high-energy virtual state relax by Raman emission to states above the Fermi energy. This requires a momentum change, that here comes from the exponential decay of optical field penetrating inside the metal, which is only strong at gap plasmon resonances as described previously^42,43^. The flares are observed at exactly these shorter wavelength gap plasmon resonances. To see why the emission becomes so much stronger within the patches than the SERS background always observed from ERS $\left( {I_{{\mathrm{bgd}}}} \right)$, we note the enhanced penetration of light into the metal at the defect patches, which gives enhanced Raman scaling as optical field to the fourth power, so that (Supplementary Note 3) the enhancement is

2 $$I_r = \frac{{I_{{\mathrm{fl}}}}}{{I_{{\mathrm{bgd}}}}} = \left( {\frac{{2a}}{w}} \right)^2\left[ {\frac{{e(\omega _p - {\mathrm{\Delta }}\omega _p)}}{{e(\omega _p)}}} \right]^5$$

Mapping the analytic flare intensity and redshift vs. (*a*, Δ*ω*_*p*_) allows us to compare with a large number of flare events (Fig. 3h, grey histograms), to estimate the characteristics of these transients. We find for this data that typically *a* = 2–3 nm and Δ*ω*_*p*_=2–3 eV, implying that such defects are indeed small but strongly perturb the local electronic properties of Au. These findings are robust to the position of the patch in the model and its shape (Supplementary Fig. 8), and we find no other model configuration which accounts for these observations (Supplementary Figs. 9–11). The patch areas are consistent with a line defect 1 nm wide stretching across the entire facet. The two broad groups I and II (Fig. 2b) show that different classes of these perturbations arise on the Au facets, while suggesting that the facet size does not control their formation (since they are not well correlated with *λ*_*C*_).

## Discussion

**Fig. 4:**
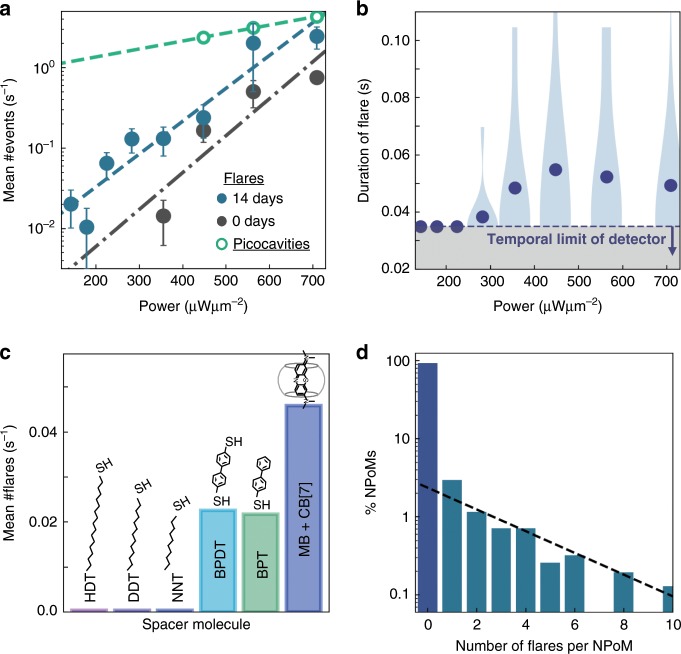
Flare dependence on illumination and molecular spacer. **a** Mean flares per second vs. power, for NPoM samples immediately after self-assembly (black) or after 14 days in air at 300 K (blue), picocavities (green). Here 500 NPoMs are surveyed for each power, lines show exponential fits, error bars indicate standard deviations. **b** Flare durations vs. power, showing mean (points) and distributions (blue violin ranges), grey region shows temporal limit of detector. **c** Occurrence frequency of flare events for different spacer molecules depicted. **d** Fraction of BPT NPoMs that show different numbers of flares, power 0.7 mWμm^−2^.

Morphological changes on the nanoscale must overcome an energy activation barrier. We show that light is responsible here by recording the power dependence of flare events. At each power ~500 NPoMs are measured over a period of 35 s (1000 SERS spectra), and the number of flare events per NPoM extracted (Fig. 4a). Exponential activation is observed which, given that only one plasmon is excited on average in the nanocavity at any one time, implies an accumulation of small structural events leads to larger scale changes. This is similarly reflected in the ‘waiting time’ before a flare event is observed after the start of laser irradiation (Supplementary Fig. 14), which exponentially accelerates at higher laser powers. Compared to picocavity activation of 0.8 eV^18^, flare activation is found to be ~5× more energetic, indeed implying that it involves multiple atomic displacements (in Mg twin-plane energies are computed to be ~1 eVnm^−2^ consistent with our estimated *a* of a few nm^44^). As the samples ‘age’ in air, the probability of flares increases (doubling every 8 days), commensurate with diffusion of individual Au adatoms at room temperature^43^. The persistence of these flares increases with laser power (Fig. 4b), showing they are metastable if energy continues to be injected (Supplementary Fig. 15). The mean flare duration saturates at 50 ms, with larger variations both within and between NPoMs.

While Au defect dynamics underlies flares, the chemical interactions between gap molecules and facet surfaces are extremely important as shown by repeating experiments with different spacer molecules within the NPoM gap in place of BPT (Fig. 4c, Supplementary Fig. 16). The DF resonance peaks confirm that the gap sizes do not change strongly with different spacers. Adding a second thiol group with 4,4’-biphenyldithiol (BPDT) has little effect on the number of flares observed per NPoM. However using alkanethiol SAMs of differing lengths (hexadecanethiol: HDT, dodecanethiol: DDT and nonanethiol: NNT) show much reduced (although nonzero) flare production rates. Instead utilising cucurbit[7]uril monolayers in the NPoM gaps in which are encased methylene blue dye molecules (MB + CB[7]) doubles the flare production rate. These results suggest the surface pinning energies of Au atoms depend on the termination bonding, on how water and ions integrate into the NPoM gap, and on the flexibility and interactions between molecules.

Our results show that localised defects can appear and migrate rapidly on gold facets (~1 nm.ms^−1^, thousand-fold faster than adatom diffusion). We suggest the most likely cause are lattice defects such as twin boundaries known to exist between crystalline domains in Au nanoparticles, allowing them to adopt geometries like decahedra and icosahedra (Fig. 1a), otherwise incompatible with bulk fcc or hcp packing^45–51^ (though defects in the Au mirror substrate could also exist). For the larger NPs here, defects are more likely and perhaps enhanced by strains induced by illumination and typically observed reconstructions^51–53^. Our transient spectra imply that facet sizes do not change (Fig. 3) while defects appear within the facets on >7% of the nanoparticles (Fig. 4d). This distribution (dashed Fig. 4d) suggests a fraction (~3%) of NPoMs are more likely to experience flare transients, though they have no obvious spectral differences (Supplementary Figs. 17–20). We mention a second possible origin of flare defects by transient small molten patches, which literature also suggests have lower conductivity^54^ hence larger Drude broadening. Such additional metal loss however does not alter the gap plasmon dispersion (Fig. 3c), nor increase the penetration into the Au (Fig. 3b), so is unable to account for the effects observed in the experiments. Local changes in *ω*_*p*_ observed on thermal roughening of Au^55^ have been irreproducible, with no understanding previously available. In addition, a number of studies show that molecules adsorbed on the surface of nanoparticles can strain the entire nanoparticle^56,57^, hence also affecting the defect production rates observed.

A key question is at what size scale does the concept of a bulk plasma frequency $\omega _p \propto \sqrt {n_e(r)/m^ \ast (r)}$ break down, and how this compares with our apparent experimental capability to measure the effective local *ω*_*p*_ on the nanoscale. Recent theory on quantum effects in plasmonics has clearly established the presence of three different regimes: a classical regime (>5 nm), a non-local regime ($\sim$1–5 nm), and a fully quantum regime below $\sim$1 nm^58^. Within the non-local regime, the comparison of the plasmonic response within fully quantum calculations with that obtained from more approximate hydrodynamical approaches has shown the validity of a mesoscopic description^59^. This modified electronic density profile from quantum calculations (which gives 0.2–0.5 nm spill-out^60,61^) is integrated into the non-local continuum approximation. The local spill-out accounts for patches in real space, and can be modelled using a thin (0.1–0.3 nm) additional surface skin on the plasmonic metal^62,63^. In small metal nanoparticles of Au or Ag, this leads to a blue-shift of the plasmon resonance, which can indeed be seen as a local modification of *ω*_*p*_, following the changes in the corresponding surface charge density profile. Consistent with this, full treatment of the screening^64^ shows that the plasma frequency can remain non-dispersive and well-defined beyond *q* > 0.2 Å^−1^ so on size scales below 0.5 nm (much smaller than we claim here). An estimate from the lateral optical field confinement in the gap $x\sim \sqrt {Dd} /n_g$ for nanoparticle diameter *D*, gap *d*, and gap refractive index $n_g\sim 1.5$, implies perturbed plasmonic lengthscales of ${\mathrm{\Delta }}x\sim {\mathrm{\Delta }}d.x/2d\sim$0.8 nm, well below the patch sizes suggested here.

We note that apart from the patch model discussed above, no other structural defect or deformation has been able to account for the effects here, including cracks, edge melting, bowing and others (Supplementary Fig. 13). While twinning planes may retain constant lattice spacing thus suggesting a uniform carrier density, other defects can provide reduced *n*_*e*_(*r*)^65,66^, although the effect on m^*^ is unknown to date. Future time-dependent DFT theory is needed to address this. Within the Drude model, our data suggests that the conductivity within the flare region drops by a ratio $\sigma _{{\mathrm{fl}}}/\sigma _{{\mathrm{Au}}} = \left( {1 - \Delta \omega _p/\omega _p} \right)^2\sim 50\%$ which thus acts as a block for local current flow in molecular electronics devices. However models do not yet account for the timescales of flickering for these defects.

The flare-enhanced ERS corresponds well to our data at short wavelengths, but it gives negligible emission near the coupled plasmon *λ*_*C*_ (Fig. 2c) because the momentum change available is too small (Supplementary Fig. 8). Despite the success of our analysis, no model of morphological defects tested accounts for the enhancement in light scattering at the coupled mode *λ*_*C*_ when illuminating simultaneously with both laser and white light (Fig. 2d). In simulations, the red-shift of the plasmon is always accompanied by a decrease in scattering peak (Supplementary Figs. 7–11). We thus now provide a tentative explanation for this nonlinear optical response based on stimulated Raman scattering (SRS) via the same metal electrons. Because the coupled plasmon mode filters input white light into a single mode, we believe that when a flare occurs, the optical confinement is sufficiently high (Supplementary Fig. 12) to generate CW SRS^67–70^ at the red-shifted coupled plasmon, even with incoherent illumination. As expected this emission is linear in both laser and white intensity. We can estimate the expected SRS enhancement for flare events as it scales as the 8th power of the field in the metal^71^ meaning a nearly 2000-fold increase due to the enhanced field penetration for flares (using scaling as in Supplementary Note 3), and assuming analogous SRS mechanisms for metallic electrons as molecules. The range of gains observed at *λ*_*c*_ varies from 100–2000% (Supplementary Fig. 18b), which shows the potential for this route to provide significant plasmonic gain in such nanocavities, of widespread interest in active plasmonics.

In conclusion, we reveal a new class of transient atomic reconfigurations at gold facets through optical confinement to the nanoscale. Analysing several million SERS spectra shows these flares are likely due to local reductions in metal plasma frequency, which increase field penetration into the metal, strongly enhancing the electronic Raman scattering. Our simplified analytical model gives excellent agreement with full electromagnetic simulations, providing the capability to examine these events in terms of defect size and local metal electronic properties. Since the longest flare events are ≤50 ms, fast optical techniques with strong plasmonic confinement and high Raman efficiencies are essential to explore them. These measurements uncover intricate cooperation between Au defect planes, chemical binding, and optical-induced forces, that are vital to account for in a wide variety of experiments, and offer new opportunities to study chemistry at metal surfaces. They suggest that strong surface restructuring occurs in which the plasma frequency can transiently vary on nm-lengthscales for ms timescales, changing electron-adsorbate interactions at the heart of applications from photovoltaics to photocatalysis.

## Methods

### Sample preparation

The Au substrates are prepared via a template stripping method, which has been detailed elsewhere^18,24–26^. Briefly, atomically smooth Au surfaces are prepared by evaporating 100 nm of Au onto Si wafers at a rate of 0.5 Ås^−1^. Small pieces of silicon are then glued to the wafer using epoxy glue, and the wafer slowly cooled from the 150 °C curing temperature to room temperature. These silicon pieces can then be peeled off to reveal a smooth Au surface with rms roughness <0.2 nm. The SAM is prepared on the Au surface by immersion in a 1 mM analyte solution in anhydrous ethanol (>99.5%) for 22 h. The nanoparticles are purchased in suspension from BBI Solutions (80 nm, OD1, citrate capped). They are dropcast onto the SAM for 30 s before being rinsed with deionised water. The short time used for dropcasting ensures a low density across the sample so they can be individually observed in optical microscopy. Aggregation is prevented by citrate capping around the Au NPs.

### Measurements

We follow millions of these emission events by examining individual NPoMs in turn for long periods of time. These NPoMs are found by particle locating algorithms which move the sample stage to select each in turn and then automatically perform the spectroscopy on each one. We remove slow stage drift using dark-field camera images to keep aligned to the location of each NPoM structure on the sample. On each NPoM structure, 1000 Raman and dark-field spectra are taken in quick succession with an integration time of 0.01 s (total read time 0.035 s).

## Supplementary information

Supplementary

Peer Review File

## Data Availability

Data for all the figures can be found at 10.17863/CAM.46892.
