# Supplementary material for: Flickering nanometre-scale disorder in a crystal lattice tracked by plasmonic flare light emission
Source: Nat Commun. 2020 Feb 3;11:682. doi: 10.1038/s41467-019-14150-w (PMC6997371; doi:10.1038/s41467-019-14150-w)
Supplement: Supplementary file 1 — Supplementary [file 41467_2019_14150_MOESM1_ESM.pdf]

Flickering nanometre-scale disorder in a crystal lattice tracked by plasmonic flare light emission

Supporting Information

Carnegie et al.

Supporting Information for Flickering nanometre-scale disorder in a crystal lattice tracked by plasmonic flare light emission

Cloudy Carnegie[1], Mattin Urbieta[2,3], Rohit Chikkaraddy[1], Bart de Nijs[1], Jack Griffiths[1], William M. Deacon[1], Marlous Kamp[1], Nerea Zabala[2,3], Javier Aizpurua[3], Jeremy J. Baumberg[1+]

[1] NanoPhotonics Centre, Cavendish Laboratory, Department of Physics, JJ Thompson Avenue, University of Cambridge, Cambridge, CB3 0HE, UK

[2] Department of Electricity and Electronics, FCT/ZTF, University of the Basque Country UPV/EHU, 48080 Bilbao, Spain

[3] Materials Physics Center CSIC-UPV/EHU and Donostia International Physics Center DIPC, Paseo Manuel de Lardizabal, 20018 Donostia-San Sebastián, Spain

Supplementary Note 1: Spectral response of NPoM to changes in the nanogap

The spectral response and near-fields of a facetted nanoparticle-on-mirror with structural changes at the facet of the nanoparticle modelled as an effective reduction in the plasma frequency, are calculated using boundary element methods (BEM) implemented in the MNPBEM Toolbox for Matlab.

The incident light angle was set at 55° (matching the experiment for dark-field scattering). A Drude model was used for the dielectric function of the gold with parameters $\varepsilon_\infty = 9.5$, $\gamma = 0.06$ eV and $\omega_p = 9$ eV, and a constant $\varepsilon_d = 2.1$ was used to model the 0.9 nm thick biphenyl-4-thiol monolayer. The nanoparticle was modelled as a truncated sphere with a radius of $R = 80$ nm on top of the monolayer, with a facet width of $w = 20$ nm. The gold substrate underneath the monolayer was modelled as an infinite region.

We model the reduction of the local electronic density n_e due to grain boundaries and lattice defects as a decrease of the local plasma frequency in regions with different geometrical shapes on top of the bottom facet of the NP. The studied cases depicted in Fig.SI15-19 include a hemi-ellipsoidal patch of different sizes and at different positions on the facet or cracks (both with a local change in ω_p keeping a constant $\varepsilon_d = 1$, and using hemi-ellipsoids with $a < b < c$), pico and nanoholes modelled as hem-ellipsoids, and toroidal patches.

Disturbances in the structure of the gap can increase the penetration of the field within the metal (Fig.3b) and change the optical response of the system in different ways. For instance, as observed in Fig.3e of the main text, increasing the lateral size of an ellipsoidal patch centred in the middle of the facet redshifts the coupled plasmon λ_c and decreases its intensity, while increasing the coupling of higher order modes and changing their intensity. For small patches ($a = b = 2$ nm, $c = 1$) the coupled plasmon shows little dependence on the height c of the ellipsoidal patch (Fig.SI16b), the position respect to the centre of the facet (Fig.SI16c) or variations of the plasma frequency ω_p (Fig.SI16d), which does not hold for higher order modes, as they are more sensitive to changes within the gap.

Similar trends are observed for the remainder of these perturbed structures, although setting the dielectric function within the patch to be $\varepsilon_d = 1$ (nanoholes) blueshifts the modes, in contrast to what

is observed in the experimental results. Moreover, fields within the nanohole do not contribute to the creation of flares, as the electron density within these is zero.

The field penetration increases within toroidal patches, as expected, and the scattering cross sections show similar trends to the ellipsoidal patches. We note shifts of the atoms at the edges of the facet are more likely to create irreversible changes to the facet size (seen previously), while the flares seen here are reversible in the experiments.

Supplementary Note 2: Derivation of analytical formula for fractional red-shift, $\delta\lambda_c/\lambda_c$

The metal-insulator-metal (MIM) dispersion, assuming a Drude model with no damping and for very thin gaps on the nanoscale, is given by [1]

$$k_{\parallel}(\omega) = -\frac{2\varepsilon_g}{d\varepsilon_m} = -\frac{2\varepsilon_g}{d}\left[\varepsilon_\infty - \frac{\omega_p^2}{\omega^2}\right]^{-1} \qquad (S1)$$

where $k_{\parallel}$ is the wavevector parallel to the surface, d is the gap thickness, $\varepsilon_g, \varepsilon_m$ are the dielectric constants in the gap and metal respectively, ω is the frequency and ω_p is the plasma frequency of the plasmonic metal cladding. Rearranging this for ω gives

$$\omega^2 = \frac{\omega_p^2}{\varepsilon_\infty + \frac{2\varepsilon_g}{k_{\parallel}d}} \qquad (S2)$$

which shows the MIM dispersion. In a uniform MIM structure which is formed under the facet of the nanoparticle, the cavity modes in this waveguide are formed subject to boundary conditions at each facet edge. This sets the allowed values of $k_{\parallel}$ since for the circular symmetry

$$k_{\parallel}w = 2\alpha_i \qquad (S3)$$

where w is the facet width and α_i are zeros of the Bessel function of the first kind. Here we work only with the lowest mode. When the facet width is fixed, this also fixes $k_{\parallel}$ so that the MIM dispersion becomes proportional to ω_p, giving rise to the dispersion shown in Fig.3c (main paper).

We now consider the situation in which the plasma frequency is not uniform along the finite MIM waveguide, but can vary. If only a small region in the MIM is perturbed, then to first order the in-plane wavevector is unchanged (from Eqn.(S3)). To calculate the shift in the lowest mode frequency, we can use first order perturbation theory, using the shape of the lowest mode as a Gaussian intensity distribution (see figure below) with a lateral FWHM[3]

$$\Delta x = \sqrt{2R\, d/\varepsilon_g} \qquad (S4)$$

given nanoparticle radius R.

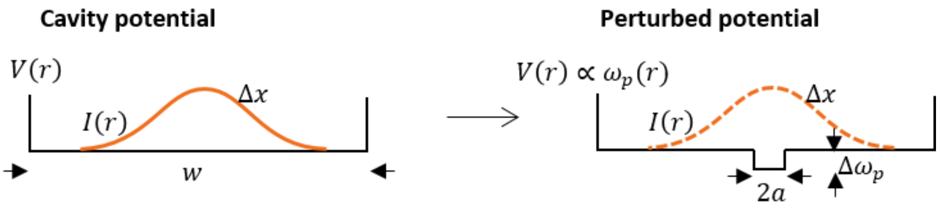

Given that the MIM plasmon from Eqn.(S2) follows $\omega \propto \omega_p$, the energy shift arises from the area integral of the cavity mode across the patch of reduced plasmon frequency (suitably normalised). The frequency shift from this perturbation is then:

$$-\frac{\delta\lambda_c}{\lambda_c} = \frac{\delta\omega}{\omega} = -\frac{1}{2}\frac{\int I(r)\frac{\delta\omega_p}{\omega_p}d^2r}{\int I(r)\, d^2r} \qquad (S5)$$

where the prefactor of a half comes from our placement of the perturbed patch on only one metal surface of the MIM cavity. Assuming an unperturbed plasmon frequency ω_{p0} we then obtain

$$\frac{\delta\lambda_c}{\lambda_c} = \frac{1}{2}\frac{\int I(r)\left[1 - \frac{\omega_p(r)}{\omega_{p0}}\right]d^2r}{\int I(r)\, d^2r}$$
$$= \frac{1}{2}\left(\frac{\Delta\omega_p}{\omega_{p0}}\right)\int_0^a \exp\left\{-\ln 2\frac{r^2}{\Delta x^2}\right\}.d^2r \qquad (S6)$$

In this approximation we obtain

$$\frac{\delta\lambda_c}{\lambda_c} = \left(\frac{\Delta\omega_p}{\omega_{p0}}\right)\frac{1}{2}\left\{1 - \exp\left(-\frac{\varepsilon_g a^2 \ln 2}{2Rd}\right)\right\} \qquad (S7)$$

Supplementary Note 3: Derivation of the intensity increase in SERS background due to flares

The displacement field perpendicular to the surface must be conserved from the dielectric gap into the metal, therefore

$$\varepsilon_m E_m = \varepsilon_g E_g \qquad (S8)$$

The penetration into the metal is given by

$$e = \left|\frac{\varepsilon_g}{\varepsilon_m}\right| \qquad (S9)$$

In the defect region ω_p is reduced, and therefore so is ε_m and the penetration into the metal (e') increases to

$$e' = \left|\frac{\varepsilon_g}{\varepsilon_m{}'}\right| \qquad (S10)$$

The skin depth $\delta_\perp$ is inversely proportional to this field penetration, $\delta_\perp \propto {}^1/_e$, because it depends on the metal permittivity.

The spontaneous electronic Raman scattering (ERS) in the metal giving the spontaneous flare is proportional to the 4th power of optical field in the metal[1], multiplied by the volume of $\pi a^2 \delta_\perp$ in which this field penetrates within the metal so that the flare intensity

$$I_{\text{fl}} \propto \pi a^2 \delta_\perp \left(eE_g\right)^4 \qquad (S11)$$

(neglecting the much smaller background contribution from the rest of the facet, see below). Assuming the Poynting flux is conserved for MIM plasmons propagating inside the gap waveguide, hence $|E_g|^2 k_\parallel$ is conserved. This leads to enhancement in the field within the dielectric at the centre

of the facet of $E_g \propto \sqrt{e'}$. This is indeed supported by the simulations: for instance reducing ω_p = 9eV to 6.5eV gives a +24% increase in E_g as predicted (Fig.S19).

The background ERS comes from the penetration of the field into the metal without the presence of the flare (using an area now $\pi w^2 \delta_\perp$). Therefore the ratio of flares to background ERS intensity is given by

$$\frac{I_{\mathrm{fl}}}{I_{\mathrm{bgd}}} = \frac{e}{e'}\left(\frac{e' e'^{1/2}}{e\ e^{1/2}}\right)^4 = \left(\frac{e'}{e}\right)^5 \qquad (S12)$$

where

$$\frac{e'}{e} = \left|\frac{\varepsilon_m}{\varepsilon_m{}'}\right| = \frac{\varepsilon_\infty - \frac{\omega_p^2}{\omega^2}}{\varepsilon_\infty - \frac{\left(\omega_p - \Delta\omega_p\right)^2}{\omega^2}} \qquad (S13)$$

A similar calculation can be performed for the stimulated Raman scattering contribution to the coupled mode, observed when illuminating simultaneously with both laser and white light, which will scale as the 8th power of optical field in the metal, multiplied by the volume within which this field penetrates, given by

$$\frac{I_{\mathrm{SRSflare}}}{I_{\mathrm{SRSbgd}}} = \left(\frac{2a}{w}\right)^2 \frac{e}{e'}\left(\frac{e' e'^{1/2}}{e\ e^{1/2}}\right)^8 = \left(\frac{2a}{w}\right)^2 \left(\frac{e'}{e}\right)^{11} \qquad (S14)$$

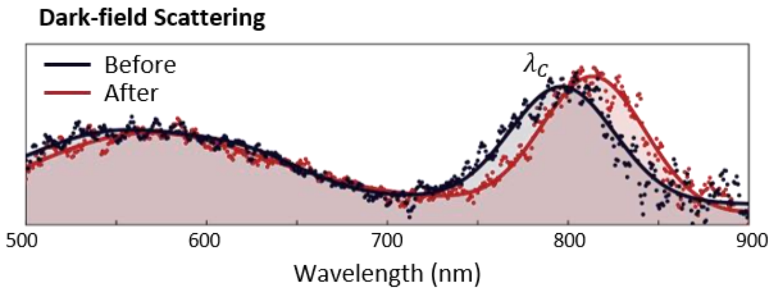

Supplementary Figure 1. Dark-field scattering spectra before and after laser irradiation. Slight redshift in coupled mode position (λ_C) indicates small amount of permanent damage to nanoparticle. Previous work has shown that this corresponds to slight increases in facet size w as atoms move from the upper NP surface down to the facet edges. [2]

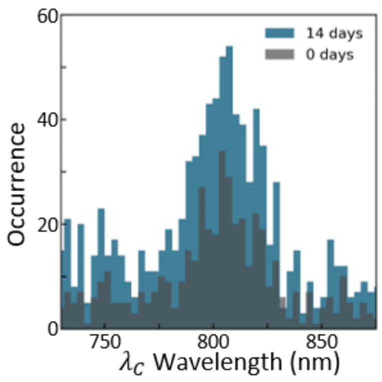

Supplementary Figure 2. Coupled plasmon mode wavelengths. Histogram showing the distribution of coupled mode wavelengths (λ_C) for both the sample at 0 days (grey) and at 14 days (blue), indicating that although there are changes to the number of flares observed, the dark-field scattering is unchanged

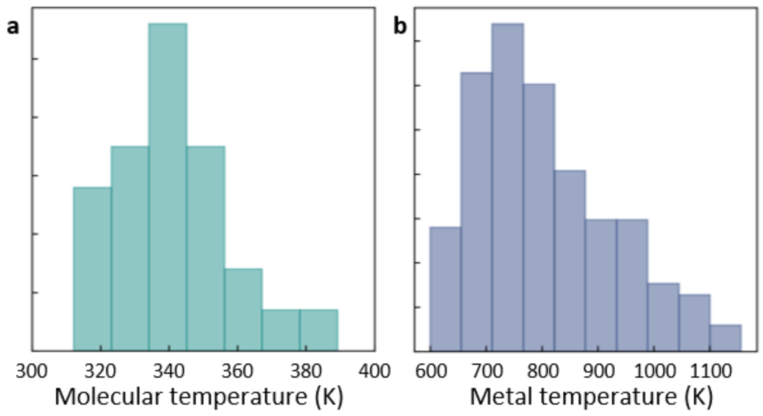

Supplementary Figure 3. Distribution of temperatures. Histograms showing the distribution of temperatures. **a.** Molecular temperatures calculated via Stokes/anti-Stokes ratios. Mean = 341±17K **b.** Apparent metal temperatures, calculated via electronic background lineshape in antiStokes emission. Mean = 734±122K. [3]

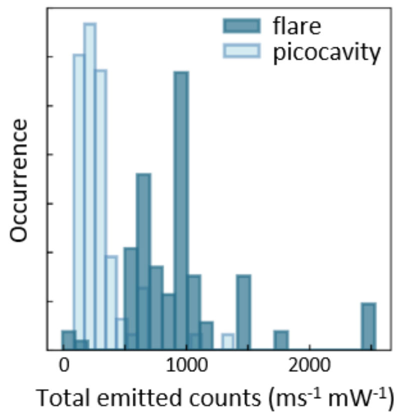

Supplementary Figure 4. Total light emission during events. Histograms of total light emission recorded during picocavity events and flare events, showing flares are 4-5-fold brighter.

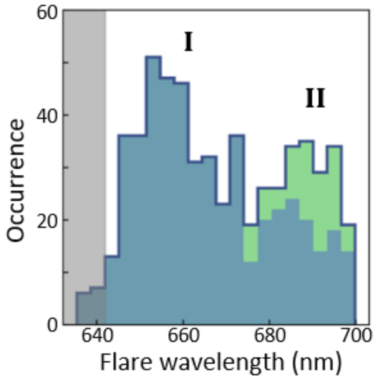

Supplementary Figure 5. Flare centre wavelengths. Stacked histogram showing the positions of flare modes recorded. Clusters I (blue) and II (green) are labelled. Grey shows filter region.

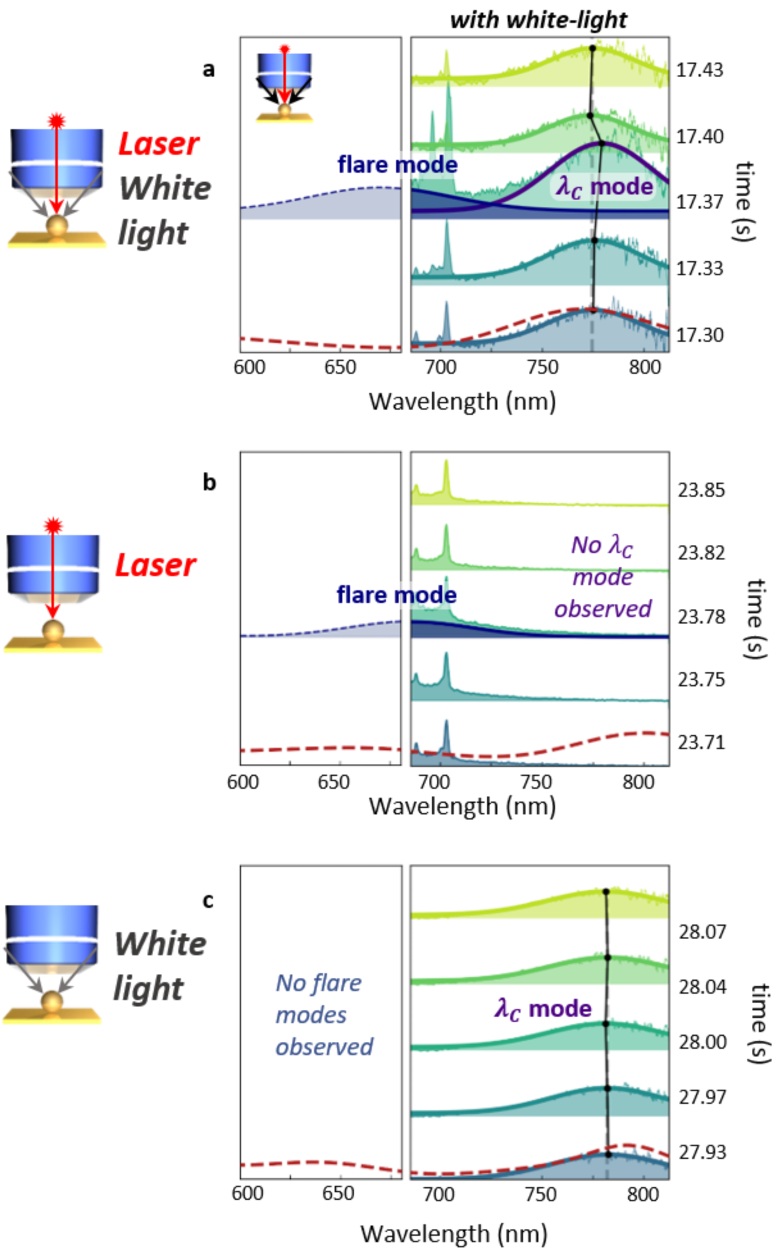

Supplementary Figure 6. Effect of simultaneous white light and laser illumination. a. When system is illuminated with both incoherent white light and 633nm laser, both coupled mode (λ_C) and flare modes can be observed. Left panel shows flare fit. **b.** Illumination with laser only enables observation of flare modes, but no coupled is visible (red dashed line shows coupled mode measured with dark-field spectrometer, measured prior to kinetic series). **c.** Conversely when illuminating solely with a white light, only the coupled mode is visible.

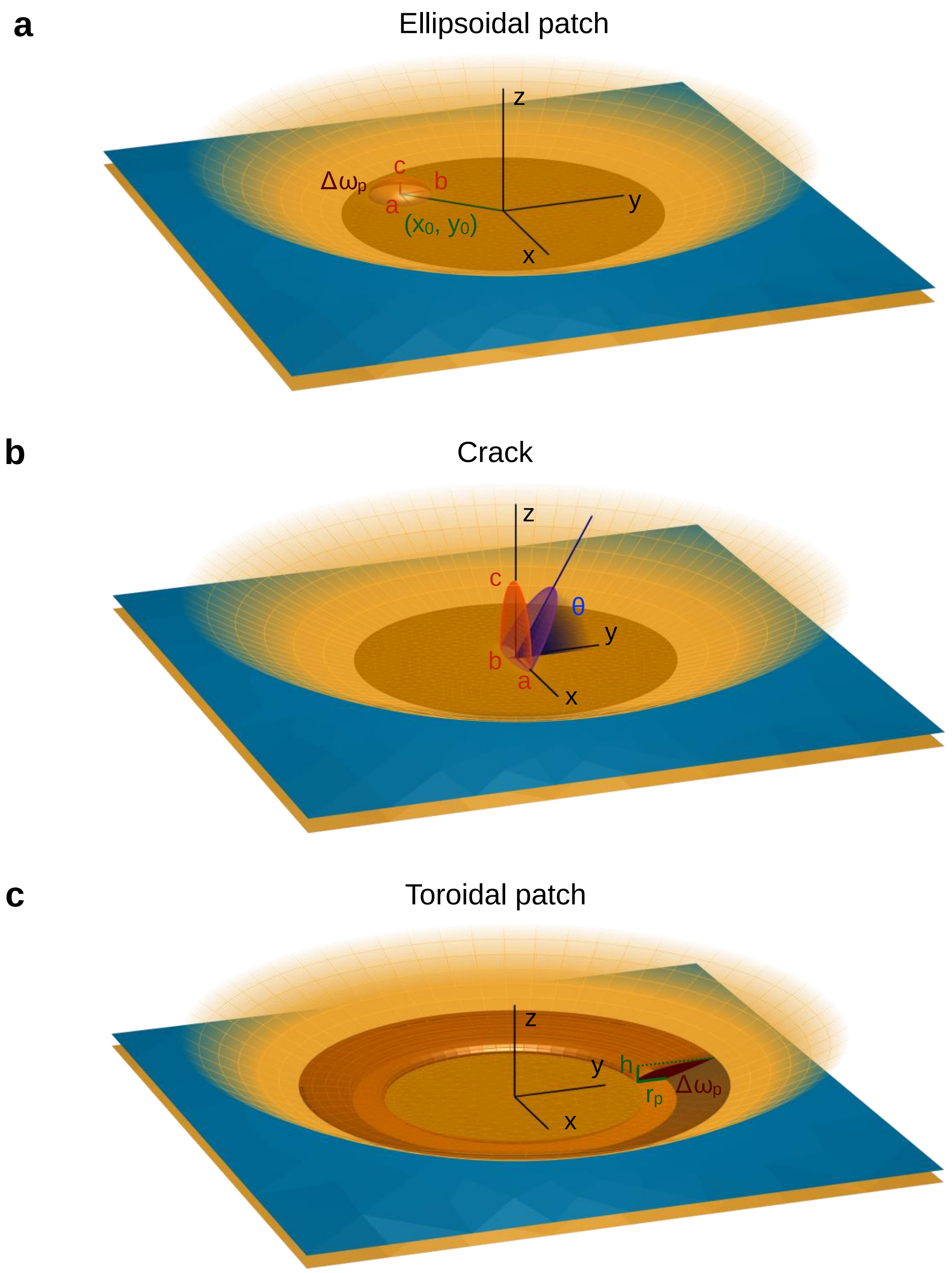

Supplementary Figure 7. Modelled crystal surface defects. a. Ellipsoidal structure used to model the patch with a change in the electron density on top of the NP facet. Patch principal semi-axes a, b and c, with its centre at position $x = x_0$ and $y = y_0$. Similar shapes are used to model the nanoholes. **b.** Vertical and tilted cracks, also modelled as ellipsoids, the tilting angle being defined respect to the xy-plane (the NP facet). **d.** Toroidal patches around the edge of the facet, with height h and radius r_p.

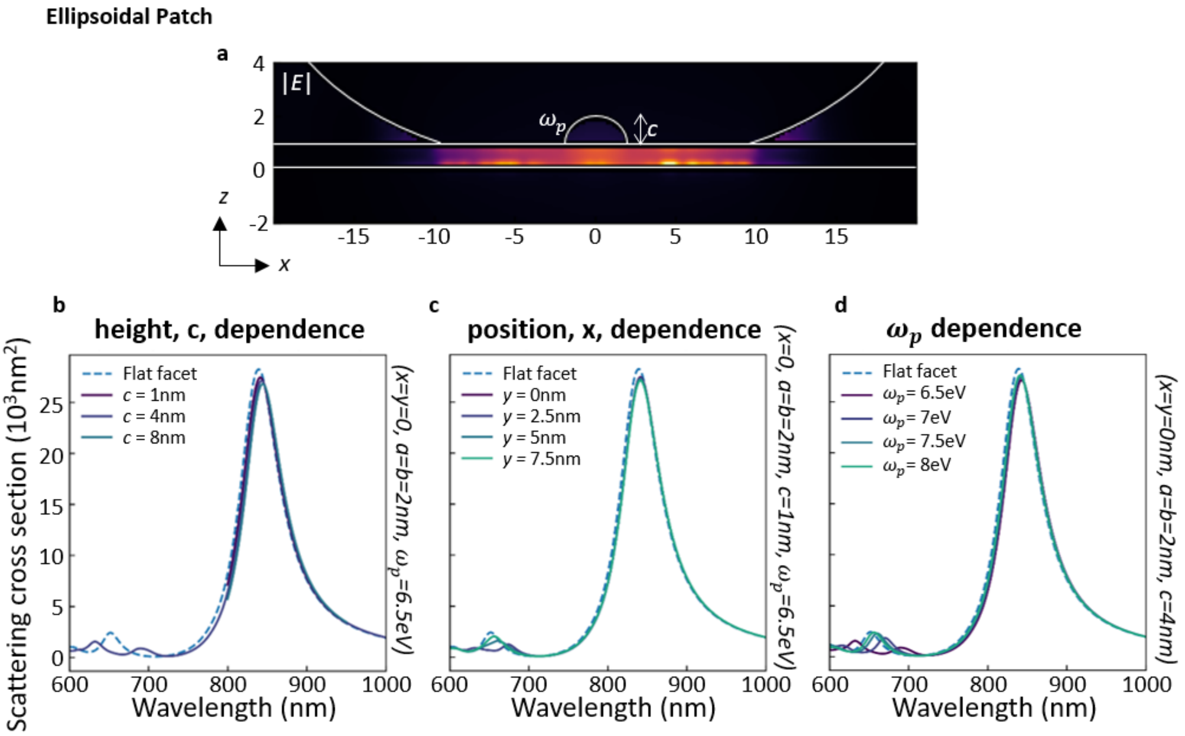

Supplementary Figure 8. Ellipsoidal patch simulations. a. Simulated field intensity around gap for ellipsoidal patch of plasma frequency $\omega_p = 6.5$eV, height c = 1nm, widths a = b = 2nm, patch position x=y=0,usingBEM. **b-d.** Dependence of calculated scattering cross section on (b) height c, (c) position x, and (d) decreased plasma frequency ω_p of patch.

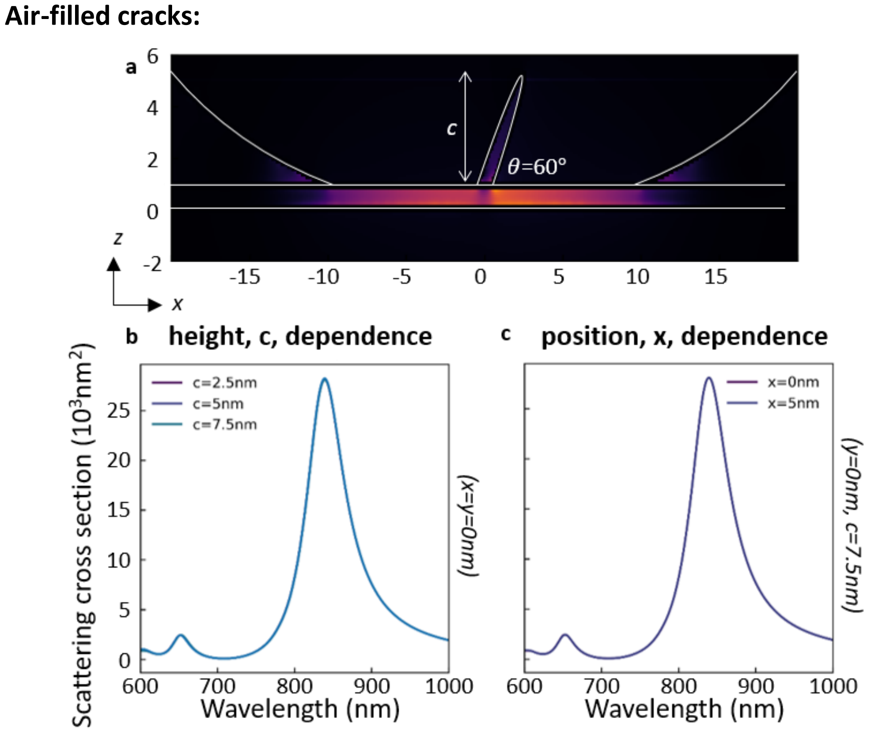

Supplementary Figure 9. Air-filled crack simulations. a. Simulated field intensity around gap for air-filled crack of height c = 6nm, crack angle to facet = 60°, position x = y = 0, using BEM. **b-c.** Dependence of calculated scattering cross section on (b) height, c, and (c) position, x.

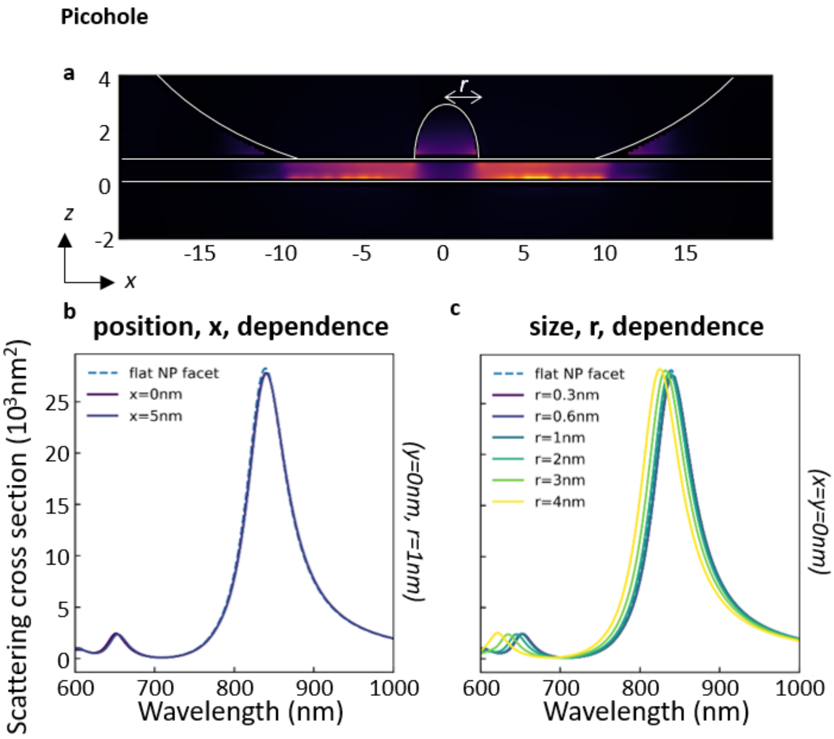

Supplementary Figure 10. Nanohole simulations. a. Simulated field intensity around gap for air-filled 'nanohole' of radius r = 1nm, position $x = y$ = 0, using BEM. **b-c.** Dependence of calculated scattering cross section on (b) position x, and (c) size of picohole of radius r.

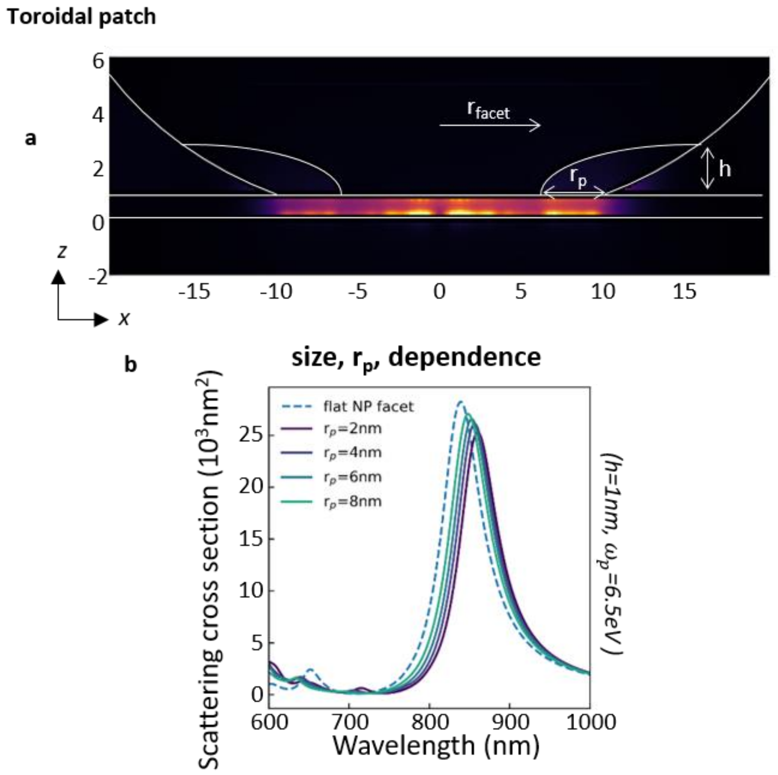

Supplementary Figure 11. Toroidal patch simulations. a. Simulated field intensity around gap for toroidal patch of radius r_p = 2nm, height h = 2nm, decreased plasma frequency ω_p = 6.5eV, and r_{facet} =8nm, using BEM. **b.** Dependence of calculated scattering cross section on radius of toroidal patch r_p.

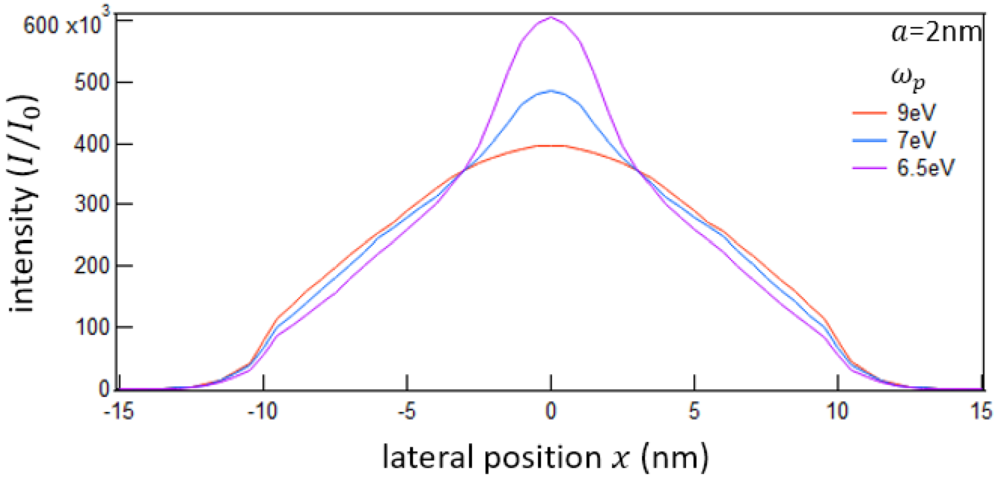

Supplementary Figure 12. Field intensity with local change in plasma frequency. Simulated intensity distribution in the centre of the gap as a function of lateral position x for a faceted NPoM with patch size a=2nm centred in the middle of the facet, and various reduction in plasma frequency from 9eV to 6.5eV.

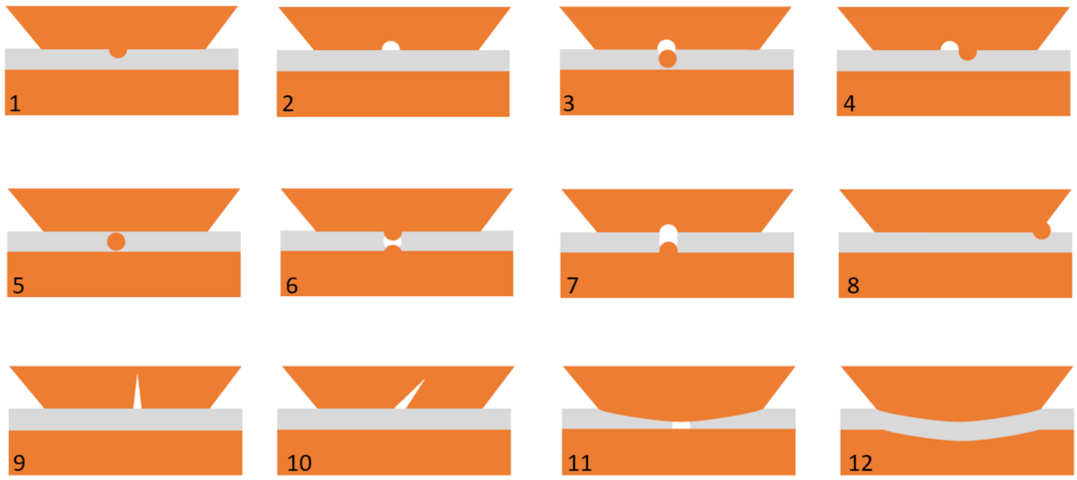

Supplementary Figure 13. Investigated gap structures for flares. Different nanoparticle-on-mirror configurations with structure in the gap investigated for modified plasmonic elastic and inelastic emission response to match data.

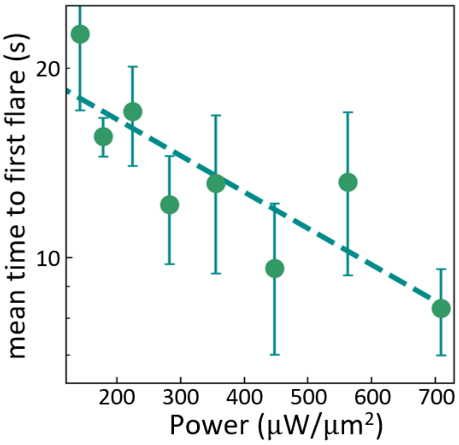

Supplementary Figure 14. Time to first flare. Mean time after laser turned on before first flare mode detected *vs* power, line shows exponential fit.

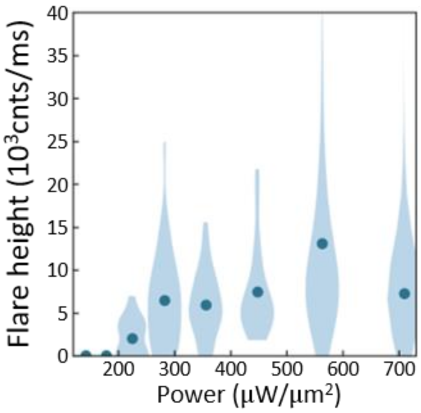

Supplementary Figure 15. Flare intensity with increasing incident power. Power series showing intensity of flares detected. Violin plots show distribution of data, points show mean values.

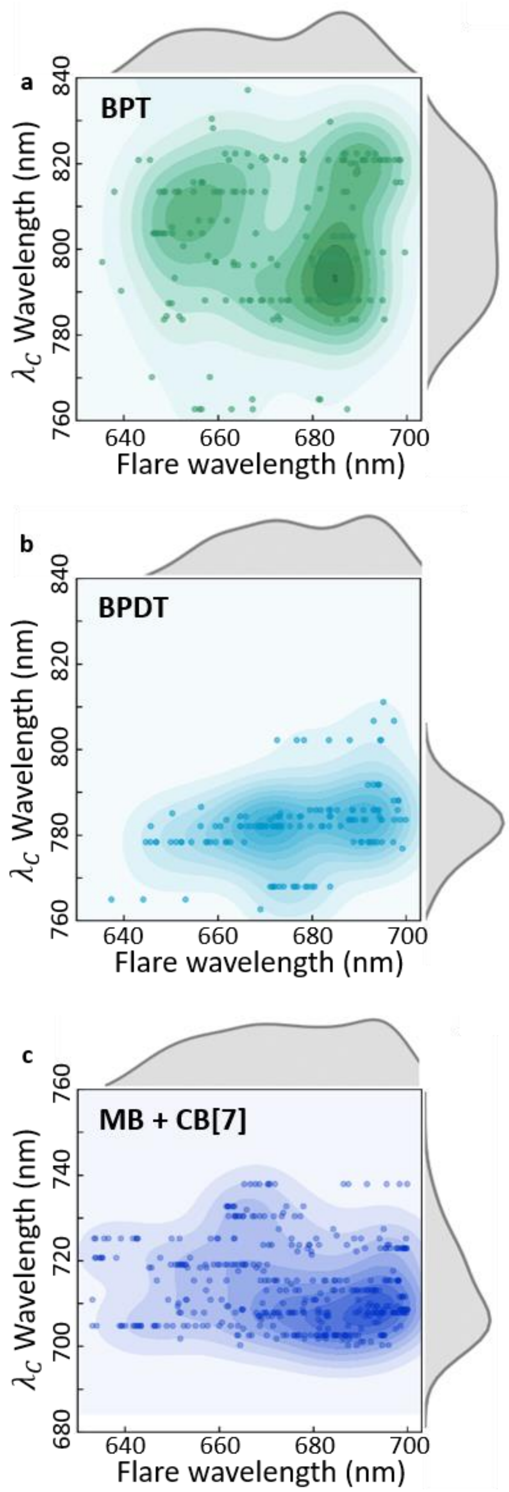

Supplementary Figure 16. Intensity maps showing spectral features of flare events observed for different molecular spacers a. biphenyl-4-thiol (BPT), **b.** biphenyl-4,2-dithiol (BPDT) and **c.** methylene blue dye (MB) encapsulated in cucurbit[7]uril molecules.

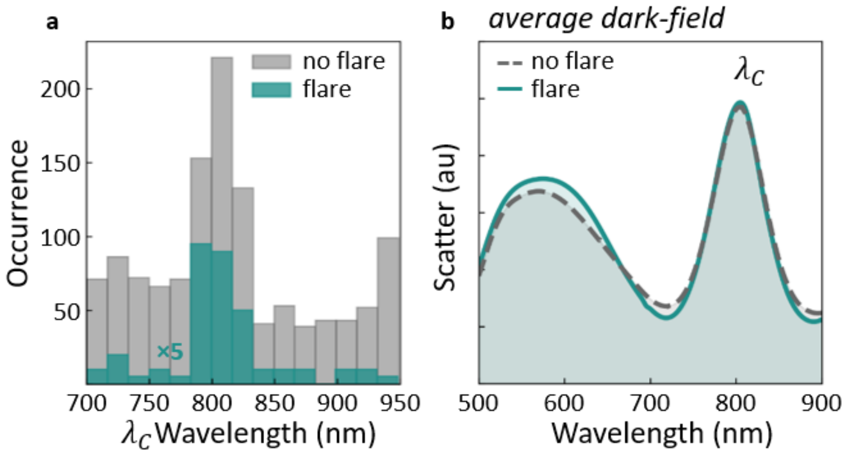

Supplementary Figure 17. Plamonic coupled mode for particles with flares. a. Distribution of coupled mode wavelengths measured via dark-field scattering. **b. A**verage scattering spectra for nanoparticles which show no flares (grey) and those which show at least one flare (green).

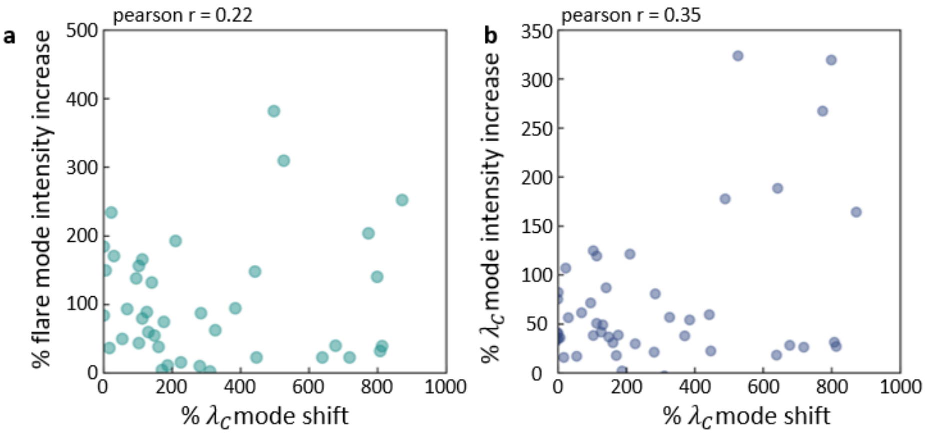

Supplementary Figure 18. Simultaneous coupled mode (λ_C) and flare mode observations. a. Correlation between increase in light intensity at flare event with simultaneously observed coupled mode shift. Pearson coefficient 0.22 indicates little correlation. **b.** Correlation between coupled mode intensity increase and coupled mode redshift, both measured simultaneously to flare event. Pearson coefficient 0.35 indicates little correlation.

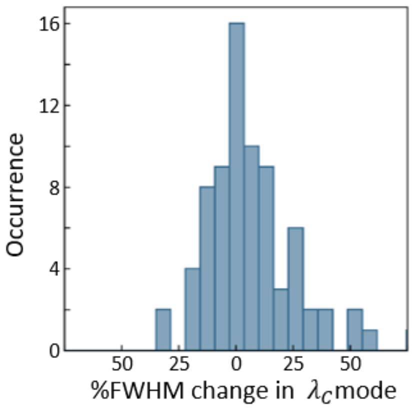

Supplementary Figure 19. Change in plasmonic coupled mode width after flare. Percentage changes in full-width-half-maximum of coupled mode from before flare to during flare, showing sometimes the coupled mode broadens and sometimes it narrows.

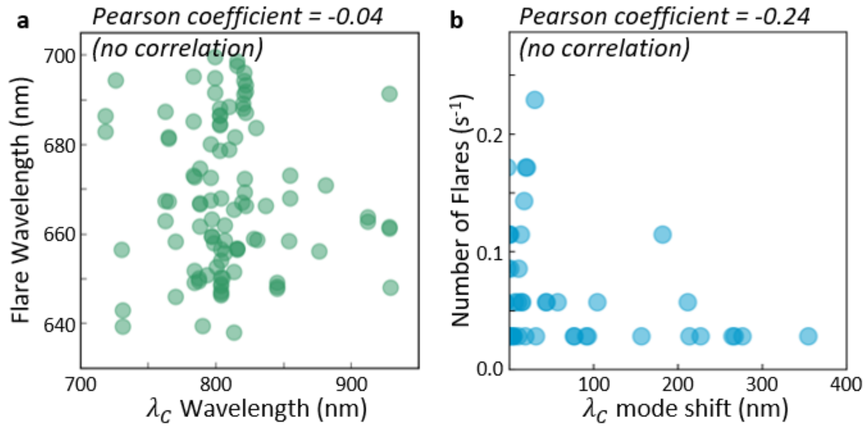

Supplementary Figure 20. Dark-field scattering measurements correlated to flare emission observations. a. Wavelength of flare mode and the corresponding coupled mode position before laser irradiation plotted against each other showing a Pearson coefficient of -0.04 (no correlation). **b.** Number of flares observed plotted against the coupled mode redshift observed for that nanoparticle before laser irradiation (same as after) showing a Pearson coefficient of -0.24 (indicating minimal correlation).

Supplementary References

[1] S. I. Bozhevolnyi and T. Søndergaard, Opt. Express **15**, 10869 (2007).

[2] J. Mertens, M.-E. Kleemann, R. Chikkaraddy, P. Narang, and J. J. Baumberg, Nano Lett. **17**, 2568 (2017).

[3] J. T. Hugall and J. J. Baumberg, Nano Lett. **15**, 2600 (2015).
