# Supplementary material for: Flickering nanometre-scale disorder in a crystal lattice tracked by plasmonic flare light emission
Source: Nat Commun. 2020 Feb 3;11:682. doi: 10.1038/s41467-019-14150-w (PMC6997371; doi:10.1038/s41467-019-14150-w)
Supplement: Supplementary file 2 — Peer Review File [file 41467_2019_14150_MOESM2_ESM.pdf]

Reviewers' Comments:

Reviewer #1:
Remarks to the Author:
The manuscript addresses the optical, near-field detection of atomic lattice scale defects in secondary optical emission spectra. The idea relies on a model calculation to clarify the relation between defects and spectra. I have a question concerning the modeling of atomic scale defects:

1. As far as I understand, to model the defects, one major parameter is the plasma frequency which is varied as a function of position to simulate the metal variation at the atomic scale. The plasma frequency however is a quantity valid at a mesoscopic scale, not below. Therefore, to me it is not clear how far this concept carries and whether it really shows that the atomic scale resolution is reached using optical fields. Even more: does the use of a mesoscopic quantity to explain the experiment not imply that a mesoscopic length scale is measured?

2. One more, but minor point (after Fig.3): The momentum introduced for the relaxation to states above the Fermi sea is probably a relative momentum between electron and hole in the Fermi sea - the momentum introduced by the spatially localized light field is the corresponding center of mass momentum - why do these different momenta fulfill the described conservation law?

In particular concerning point 1 I cannot recommend the manuscript for publication in its present form.

Reviewer #2:
Remarks to the Author:
Carnegie et al. describe the observation of relatively rare "flare" light emissions from a Au - SAM - AuNP system, using purely optical means to detect these. They model these flares by transient defects in the AuNPs that give rise to (i) a shift in the plasmon resonance, and (ii) the occurrence of the flares.

The manuscript is generally well written, and the modeling / theoretical explanations appear to capture the essential features of the flares. My main issue when reading the manuscript was that it is not very accessible the way it is written, extremely narrow in scope, aimed only at a very small number of highly specialized researchers. The authors make little effort in the manuscript to put their findings into a broader context (the exception being a few vague hints in the introductory paragraph). For example, what are the implications for nanoparticle (photo)catalysis? or for nanospectroscopy such as TERS? or other fields? I would encourage the authors to at least speculate in this direction (and declare speculations as such in the text); this will immediately create much more interest in the broader scientific community.

A second, important point is that much more detail should be given about the experimental approach, at the moment this is very unclear. How was the Au single crystal prepared, kept clean, and covered with the SAM? How were the NPs spread onto the SAM? What was their density? How was aggregation of NPs prevented? What exactly was the optical scheme to follow millions of emission events? Why was tracking necessary, did the particles move around or is this an issue of drift? What was the integration time / time resolution of the optical detection?

Moreover, there are a few minor issues that should be addressed / questions that should be answered in the text:
- I kept on asking myself whether dynamic defects in the underlying Au could lead to the same observations
- the dependence on the SAM molecules (results in Fig. 4c) is not well explained. For example, the alkane thiols could bend over, leaving essentially a similar distance as the biphenyl SAM between

the Au surface and the NP layer.
- I also find the statements on the following page about how pinning energies of Au atoms depend on the chemical environment vague and not well justified by the modeling.
- what is a "toy model" supposed to be?
- is the modeling capturing the timing of the flare events in any way? If not, this should be clearly stated.

Reviewer #1

1. As far as I understand, to model the defects, one major parameter is the plasma frequency which is varied as a function of position to simulate the metal variation at the atomic scale. The plasma frequency however is a quantity valid at a mesoscopic scale, not below. Therefore, to me it is not clear how far this concept carries and whether it really shows that the atomic scale resolution is reached using optical fields. Even more: does the use of a mesoscopic quantity to explain the experiment not imply that a mesoscopic length scale is measured?

> The referee raises a really crucial point, which highlights the wider scale interest of our paper. Firstly we note that the explanation we have come to here has only emerged after trying an enormous number of different possible models which we have rigorously simulated (some are described in detail in the SI), as seen in Fig.R1 below.

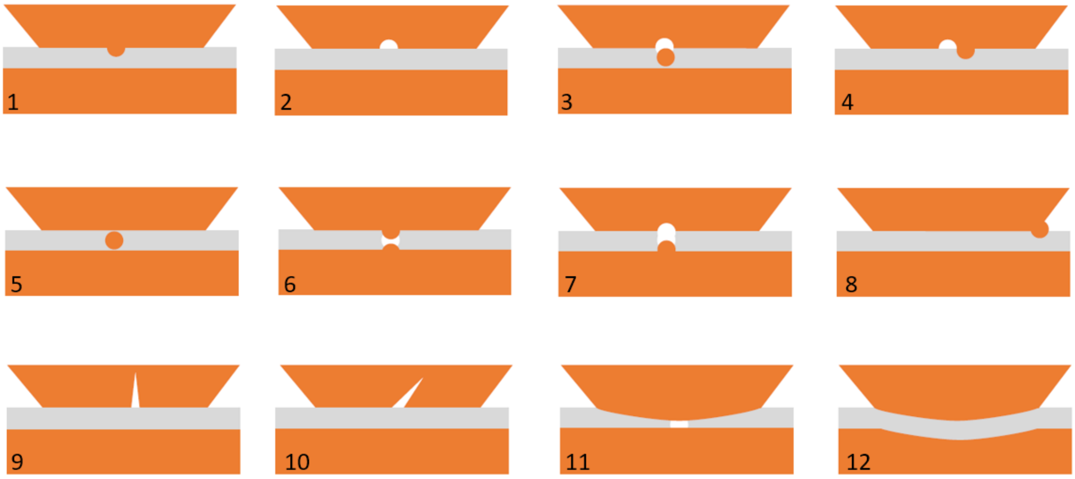

Fig.R1: Different nanoparticle-on-mirror configurations with structure in the gap investigated for modified plasmonic elastic and inelastic emission response to match data.

Despite our similar misgivings to the referee, the only configuration which is able to account for almost all of the experimental observations, is the one put forward in the paper. Essentially the difficulty is that configurations with holes, asperities, or voids all produce local enhancements in optical field within the gap (similar to picocavities, see SI Figs.15-20) which would strongly increase the molecular SERS signal, but which is not experimentally seen. Instead, the observations point to an enhancement of light entering the metal facets which is expressed by changes in the dielectric contrast. The unaffected SERS peaks unambiguously shows that this is not from changes in the molecules inside the gap, but must be from the metal.

In the Drude continuum model, metal permittivity changes are attributed to changes in the plasma frequency (the damping is shown in the SI not to have much effect on the resonance positions), but we fully agree that this is a continuum approximation to the full quantum situation, Since the legitimacy of our approach is of crucial importance, as the referee points out, we now consider this in more detail in the following response, as well as in the manuscript.

It is already clear that the classical ω_p has more nuanced contributions from internal band structure since the Drude parameters change abruptly when bulk Au is melted, despite the similar atomic spacing. The referee rightly emphasises our use of a mesoscopic approximation to define ω_p on the nanometre scale (we are careful not to specify 'atomic scale' for variations in ω_p). Indeed our continuum model shows that the only way to fit our data is to assume facet patches of around 5nm have a reduced ω_p (more than ten times the atomic spacing). In the following we point different contexts where such an approach has been extensively used to address the optical response of metals within the 1-to-5 nm scale.

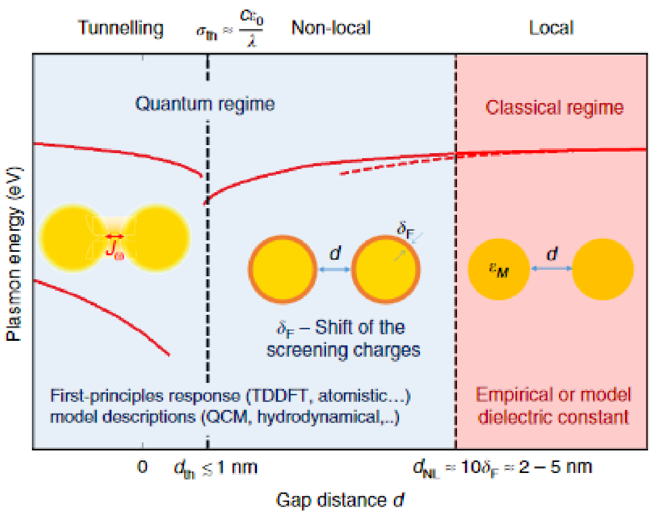

Fig.R2: Progression through plasmonics regimes from classical (d>5nm) to atomic (d<1nm) through the mesoscopic 'non-local' quantum regime. Figure is from Zhu et al. Nature Comm. ***7****, 11495 (2016).*

Recent theoretical work on quantum effects in plasmonics, including work by some of us and others, has clearly established the presence of three different regimes of response in plasmonics: a classical regime (> 5 nm), a non-local regime (1 nm to 5 nm), and a fully quantum regime below 1 nm (see Fig. R2 for details in a recent review of ours). Within the non-local regime, the comparison of the plasmonic response within fully quantum calculations with that obtained from more approximate hydrodynamical approaches has set the validity of such a mesoscopic description This is explicitly shown in the work from the Mortensen group (see Nature Comm. **6**, 7132 (2014) *Resonance shifts and spill-out effects in self-consistent hydrodynamic nanoplasmonics*) which uses the electronic density profile (spill-out) from quantum calculations integrated into the non-local continuum approximation. Indeed, in small metal nanoparticles of Au or Ag, this leads to a blue-shift of the plasmon resonance, which can be similarly thought of as a local modification of ω_p in real space, following changes in charge density profiles. Despite this attribution, it is not yet clear how one would divide this into changes in electron density and changes in effective mass, which are the ingredients of ω_p. Consistent with the use of effective models in this mesoscopic regime, a full treatment of the screening [see Pitarke et al, Rep.Prog.Phys **70**, 1 (2007) for a review] shows that the plasma frequency remains non-dispersive and well-defined beyond q>0.2Å^{-1} so on size scales below 0.5nm (much smaller than we are claiming here).

Through decades of surface physics, there have indeed been many studies which nicely applied the effect of a 'modified' electronic density in this 'spill-out', by adopting an effective dielectric function which follows the density profile in the corresponding patches in real space. This can be observed for example in A.D. Boardman et al. (Surf. Sci. 1975), F. Forstmann, H. Steschke (Phys. Rev. B, 1978), and P. Ahlqvist, P. Apell (Phys. Scr. 1982). Furthermore, very recently, along the lines of this real-space effective approach in the mesoscale, TDDFT shows that the effect of non-locality can successfully be modelled as a thin additional surface skin on the plasmonic metal [PRL **111**, 093901 (2013) and PRL **110**, 263901 (2013)] of dimensions 0.1-0.3nm, which gives one of the length-scales that the referee asks about.

To estimate from this a lateral lengthscale on which it makes sense to define a mesoscopic ω_p we note that lateral optical field confinement in the gap scales as $x{\sim}\sqrt{Dd}/n_g$ for nanoparticle diameter D, gap d, and gap refractive index n_g~1.5. The non-local plasmonic response thus corresponds to a lateral scale $\Delta x{\sim}\Delta d.x/2d$~0.8nm which suggests that the approximations used for the model in the paper are not inappropriate.

Finally, one of our aims in this work is to point to careful extensive well-characterised experimental data which points out the need from further theoretical advances in this area, bringing quantum mechanics and classical electrodynamics together. We thus give a tentative model in the continuum approximation, supported by previous literature and by our own findings on its validity, while aiming to stimulate strong theory developments. To encourage this, we extend considerably the discussion at the end of the paper, raising explicitly the comments of the referee in the context of the previous work noted here.

2. One more, but minor point (after Fig.3): The momentum introduced for the relaxation to states above the Fermi sea is probably a relative momentum between electron and hole in the Fermi sea - the momentum introduced by the spatially localized light field is the corresponding center of mass momentum - why do these different momenta fulfill the described conservation law?
> Since it is the single-electron bandstructure considered here (see ref [44]), the momentum is relative to the ionic lattice and is thus consistent with momentum conservation laws.

Reviewer #2
1. My main issue when reading the manuscript was that it is not very accessible the way it is written, extremely narrow in scope, aimed only at a very small number of highly specialized researchers. The authors make little effort in the manuscript to put their findings into a broader context (the exception being a few vague hints in the introductory paragraph). For example, what are the implications for nanoparticle (photo)catalysis? or for nanospectroscopy such as TERS? or other fields? I would encourage the authors to at least speculate in this direction (and declare speculations as such in the text); this will immediately create much more interest in the broader scientific community.
> While we are not strongly in favour of speculation, we extend the introduction and discussion as suggested, to introduce some of the implications in more detail.

2. A second, important point is that much more detail should be given about the experimental approach, at the moment this is very unclear. How was the Au single crystal prepared, kept clean, and covered with the SAM? How were the NPs spread onto the SAM? What was their density? How was aggregation of NPs prevented? What exactly was the optical scheme to follow millions of emission events? Why was tracking necessary, did the particles move around or is this an issue of drift? What was the integration time / time resolution of the optical detection?
> These are helpful clarifications now added to the manuscript. The Au substrate was prepared via a template stripping method, which has been detailed elsewhere. Briefly, atomically smooth Au surfaces are prepared by evaporating 100 nm of Au onto Si wafers at a rate of 0.5 Å/s. Small pieces of silicon are then glued to the wafer using epoxy glue, and the wafer slowly cooled from the 150°C curing temperature to room temperature. These silicon pieces can then be peeled off to reveal a smooth Au surface with rms roughness <0.2nm. The SAM is prepared on the Au surface by immersion in a 1mM analyte solution in anhydrous ethanol (>99.5%) for 22h. The nanoparticles are purchased in suspension from BBI Solutions (80nm, OD1, citrate capped). They are dropcast onto the SAM for 30s before being rinsed with deionised water. The short time used for dropcasting ensures a low density across the sample so they can be individually observed in optical microscopy. Aggregation is prevented by citrate capping around the Au NPs (during synthesis at BBI).

We follow millions of these emission events by examining individual NPoMs in turn for long periods of time. These NPoMs do not diffuse, but our 'particle tracking' (confused the referee so now changed to 'particle locating') algorithms move the sample stage to select each in turn and then automatically perform the spectroscopy on each one. We remove slow stage drift using dark-field camera images to keep aligned to the location of each NPoM structure on the sample. On each NPoM structure, 1000 Raman and dark-field spectra are taken in quick succession with an integration time of 0.01s (total read time 0.035s).

3. I kept on asking myself whether dynamic defects in the underlying Au could lead to the same observations
> The referee is correct that defects in the underlying Au could also be responsible. However it is less clear why defects would flicker around, and Au NPs (Fig.1a) are known to support twinning planes resulting from their non-bulk crystal structures (ie icosahedra, pentagonal bipyramids, etc). This is an important suggestion, so we add this note to the manuscript.

4. The dependence on the SAM molecules (results in Fig. 4c) is not well explained. For example, the alkane thiols could bend over, leaving essentially a similar distance as the biphenyl SAM between the Au surface and the NP layer.

> The nominal alkanethiol length in the gap is not very different from the biphenyl SAM. This is easily confirmed from our dark-field spectroscopy which gives a coupled plasmon resonance that indicates the gap thickness. Hence the difference for the rate of flares produced in Fig.4c is not from the gap thickness. This is now explicitly discussed.

5. I also find the statements on the following page about how pinning energies of Au atoms depend on the chemical environment vague and not well justified by the modeling.

> Connected to #4 above, the observed differences in flare production rates between different molecules attached to the Au are not yet explained. All we do is point out here that the effect is extremely strong, and suggest it is related to pinning. Indeed we did not attempt to model this, as many different results can be obtained from DFT of thiol-Au attachment, and instead encourage DFT to use our results as an input.

6. What is a "toy model" supposed to be?

> Our model is necessarily simplistic (see comments of referee 1), but constrains the range of explanations that we possible. We removed its description as a ‘toy model’ which confused this reviewer.

7. Is the modeling capturing the timing of the flare events in any way? If not, this should be clearly stated.

> While our model uses observations to constrain the spatial scales of the flickering defects we observe, it does not constrain the timing. As suggested, we thus add a comment to the manuscript.

Reviewers' Comments:

Reviewer #1:
Remarks to the Author:
I have read carefully the reply to the referees, in particular to my comment on the applicabílity of a mesoscopic approach.
The paper has clearly improved: the ideas of the authors are now more easy to read and to follow, also accompanied by the corresponding references. I still insist that the whole idea is speculativ, the proof is not direct and relies more like an exclusion of different other effects, which might not be all what can be thought of.
However, even taking this into account I am willing not to object against publication of the proposed nm-scale disorder detection by plasmonic light emission to provide the ideas for a discussion within to community. But this is really a borderline decision, leaving me not fully satisfied.

Manuscript ID: NCOMMS-19-13829A
Flickering nanometre-scale disorder in a crystal lattice tracked by plasmonic flare light emission

Response to Reviewers

Reviewer #1

"I have read carefully the reply to the referees, in particular to my comment on the applicabílity of a mesoscopic approach. The paper has clearly improved: the ideas of the authors are now more easy to read and to follow, also accompanied by the corresponding references. I still insist that the whole idea is speculativ, the proof is not direct and relies more like an exclusion of different other effects, which might not be all what can be thought of.

However, even taking this into account I am willing not to object against publication of the proposed nm-scale disorder detection by plasmonic light emission to provide the ideas for a discussion within to community. But this is really a borderline decision, leaving me not fully satisfied."

We are very happy that Reviewer #1 agrees with the improvements in clarity within the paper, and that this work will lead to valuable discussion within the community.
